# New Perspectives on Antimicrobial Agents: Rezafungin

**DOI:** 10.1128/aac.00646-23

**Published:** 2024-12-12

**Authors:** Nicholas M. Forrister, Todd P. McCarty, Peter G. Pappas

**Affiliations:** 1 Division of Infectious Diseases, Department of Medicine, University of Alabama at Birmingham 155591 , Birmingham, Alabama, USA; 2 Birmingham Veterans Affairs Medical Center 19957 , Birmingham, Alabama, USA; University of Pittsburgh School of Medicine, Pittsburgh, Pennsylvania, USA

**Keywords:** candidemia, invasive candidiasis, echinocandin, anti-fungal agents

## Abstract

Candidemia and invasive candidiasis persist as significant causes of morbidity and mortality. As fluconazole resistance rates rise, alternative means of treatment are necessary, either via mold-active azoles or extended durations of echinocandins. These come with the potential for undesirable side effects for triazoles or choosing between prolonged hospitalization or outpatient parenteral antimicrobial therapy in the case of echinocandins. Rezafungin offers an opportunity to manage extended treatment durations with shorter hospital stays and no extended parenteral access. Herein, we review the most recent published data pertaining to rezafungin including anti-fungal activity, pharmacokinetics, and clinical data relating to the treatment of invasive candidiasis. Given its prolonged half-life allowing for once-weekly dosing, rezafungin has the potential as an anti-fungal prophylactic agent in high-risk patients, and studies to examine this potential role are ongoing.

## PERSPECTIVE

Candidemia and invasive candidiasis (IC) remain challenging infections with morbidity and mortality estimated around 40% (1). These infections are commonly associated with healthcare exposure, especially in intensive care settings (2). Echinocandins have been a cornerstone of therapy in treating candidemia and invasive candidiasis demonstrating improved survival compared with other anti-fungal agents (3). Echinocandins act via inhibition of the enzyme 1,3-beta-D glucan synthetase enzyme by binding its FKS catalytic subunits, preventing the production of this crucial component of the fungal cell wall (4). The fungal cell wall loses structure leading to osmotic instability and cell death (5). This sequence allows echinocandins a broad spectrum of activity against *Candida* species. Minimum inhibitory concentration (MIC) for echinocandins against most species of *Candida* is low, including the triazole-resistant *C. krusei* and *C. glabrata*. MICs are slightly higher for *C. parapsilosis* compared to other *Candida* species, but this is of unclear clinical significance (2). Beta-D glucan is not found in animal cell walls affording this class a low side effect burden, unlike other anti-fungal agents (4). Only three echinocandins, caspofungin (CAS), micafungin, and anidulafungin, were previously available for clinical use. These drugs are semi-synthetic derivatives of natural products, the first of which was discovered in *Aspergillus nidulans* in 1974 (5). Prior studies have not shown significant differences in efficacy among these first-generation echinocandins (6). However, the use of echinocandins has also been associated with prolonged hospitalization necessitated by continued parental therapy (7). Despite their overall utility, treatment failure occurs still in 30%–40% of cases (3, 8). Treatment failure is common in the critically ill. Studies have shown that penetration differs depending on body weight and in those with critical illness (9). Additionally, resistance patterns are evolving in *Candida* species, and the increasing usage of immunosuppressive agents makes the drug-drug interactions of azole class anti-fungal challenging. The prevalence of *Candida auris* infections has been on the rise and demonstrates substantial resistance to current anti-fungal agents. Mutations in the genes encoding FKS subunits, which have been shown to confer resistance to all echinocandins, are also of great concern (5). Recently, the FDA approved the novel echinocandin, rezafungin (RZF). This medication acts similarly to other drugs of its class, while adding unique pharmacologic properties that may help address some of the challenges in treating candidemia and IC (8, 10). Two clinical trials have been conducted to evaluate its efficacy, safety, and tolerability compared to caspofungin for treating candidemia and invasive candidiasis.

## BACKGROUND

Rezafungin is a semi-synthetic echinocandin derived from anidulafungin and developed with unique pharmacokinetic properties. Its chemical structure has been altered to confer a significantly longer half-life than other echinocandins, around 133 hours (8). It retains many of the common facets of the class of medications. It is only available in intravenous (IV) formulation with excellent side effects and safety profiles (8, 10). Similar to other compounds in the class, there are no significant drug-drug interactions (11). An evaluation of nine drugs with known CYP interactions was conducted with concomitant RZF use and no relevant pharmacokinetic events occurred (12). RZF is non-renally excreted and does not require dose adjustment for renal failure based on phase I data (13). It does not affect the QTc in healthy subjects (14). Hepatic dose adjustments do not appear to be required (15). CLSI breakpoints were recently agreed upon. Rezafungin MIC susceptible-only breakpoints were listed for *C. albicans* (≤0.25), *C. auris* (≤0.5), *C. dubliniensis* (≤0.12), *C. glabrata* (≤0.5), *C. krusei* (≤0.25), *C. parapsilosis* (≤2), and *C. tropicalis* (≤0.25) (16).

## CLINICAL DATA

Phase II and phase III trials evaluating utility in candidemia and IC have been completed for rezafungin. STRIVE was the phase II, double-blind, randomized control trial (RCT) comparing RZF to the safety and efficacy of caspofungin (8). Patients included were adults with signs of infection and evidence of candidemia or IC. Those with prosthetic joint infections, septic arthritis, central nervous system (CNS) infections, eye infections, neutropenia, elevated transaminases, and Child-Pugh >9 hepatic impairment were excluded from this trial. Patients were randomized to one of three groups: RZF 400 mg once weekly, RZF 400 mg on week 1 followed by 200 mg weekly (400/200), or CAS with the ability to step down to fluconazole daily over the study period. Primary endpoints were the resolution of signs of symptoms at day 14 and mycological eradication. Secondary endpoints were all-cause mortality, clinical response at 5, 14, and 28 days. Safety data were also collected. The intention-to-treat analysis included 207 patients with a median treatment length of 14 days. The microbiological intention-to-treat analysis, those with documented *Candida* infection, included 183 subjects. Overall cure was 60.5% for RZF 400, 76.1% for RZF 400/200, and 67.2% for CAS. All-cause mortality was 15.8% for RZF 400, 4.4% for RZP 400/200, and 13.1% for CAS. Interestingly, at 5 days, the RZF pooled group had a cure rate of 62.3% compared to 55.7% in the CAS group. Negative blood cultures were obtained faster in the RZF group by 3.3 hours which was statistically significant (*post hoc P* = 0.02). RZF and CAS had equivalent cure rates among different *Candida* species. Side effects were mild and included fever, gastrointestinal (GI) upset, and hypokalemia, which were not significantly different between groups. The difference in outcomes between the RZF 400/200 and 400 groups was attributed to the large number of indeterminate results in the 400-only group and was not felt to be related to the overall efficacy, safety, or tolerability of the different doses. Reasons for failure were not significantly related to tolerability or safety events. Even when factoring out indeterminate patients, the 400/200 group outperformed the other two arms. The authors argue the Eagle effect, the paradoxical microbial growth precipitated by high antimicrobial levels, has not been reproducible in clinical trials and does not explain the apparent differences in treatment groups. Additionally, this difference was noted at day 5, while the dose in both groups was still 400 mg.

The definitive phase III study was a double-blind, RCT evaluating RZF 400/200 versus standard treatment with CAS (ReSTORE) (10). Exclusion criteria were almost identical to STRIVE; however, patients with neutropenia were allowed to be included in ReSTORE. One hundred ninety-nine patients were randomized. The main endpoints were clinical, mycologic, or radiologic cure at 14 days for the European Medical Agency and 30-day all-cause mortality for the Food and Drug Administration. This trial set a non-inferiority margin of 20%. Median therapy duration was again 14 days. Cure rates were 59% for RZF and 61% for CAS (treatment difference 95% CI: −14.9 to 12.7). All-cause mortality rates were 24% in the RZF group and 21% in the CAS group (treatment difference 95% CI: −9.7% to 14.4%). Clinical and mycologic reasons for failure were similar between groups and met non-inferiority criteria for both primary endpoints. Safety outcomes in this study were similar to the data from STRIVE as there was no significant difference between the groups in ReSTORE. The most common adverse effects were pyrexia and hypokalemia. Pooled analysis of both studies demonstrated similar safety profiles as well (17). Early treatment differences in mycologic response and global clinical picture occurred at day 5 in the RZF group, but these differences normalized at day 14. A similar trend was noted in the STRIVE study, with a time to negative culture of 23.9 hours for RZF and 27 hours for CAS, but this finding was not statistically significant. This early treatment benefit was echoed when STRIVE and ReSTORE data were pooled (17). Additional analysis of outcomes based on *Candida* species in ReSTORE was conducted (18). Breakdown of species was similar between the RZF and CAS groups. Rates of mycologic cure and 30-day mortality were comparable independent of species. Overall rezafungin was shown in these trials to be non-inferior to caspofungin in treating candidemia and IC (8, 10). Side effect profiles and toxicity levels were similar between both drugs in each study. In both trials, patients with hepatic failure were excluded. These patients were excluded from STRIVE and ReSTORE given the lack of caspofungin data on use in hepatic impairment. A 2021 evaluation of RZF in hepatic impairment showed a reduction in overall drug exposure via area under the concentration–time curve (AUC) compared to healthy patients and equivocal half-life (15). Adverse events were similar between both agents and had no significant worsening of outcomes.

## POTENTIAL ROLES FOR CLINICAL USE

Rezafungin’s extended half-life and broad spectrum of activity confer a host of potential clinical benefits. In patients with candidemia who are clinically improving, rezafungin could shorten hospital stays. RZF could negate the need for continued hospitalization or the need for the establishment of intravenous outpatient therapy. An echinocandin stewardship analysis found around 30% of patients hospitalized for candidemia were on an echinocandin on the day of discharge, but only between 6% and 15% were discharged on an echinocandin (19). This finding suggests delays in potential for discharge due to the need for extended IV therapy. Furthermore, in ReSTORE, 17% of patients in the RZF group and 28% of patients in the CAS group required 15–28 days of therapy (10). This difference represents only 1–2 more doses of RZF as opposed to up to 14 more daily doses of CAS. This benefit would better utilize healthcare resources but also could improve patient safety by reducing the total burden of healthcare interventions. More complex cases of candidiasis such as endocarditis and osteomyelitis, which typically require extended duration of therapy, can complicate management decisions. Intravenous catheter placement to expedite discharge can be risky, given *Candida* species predisposition to infect indwelling vascular catheters. The once-weekly dosing schedule could negate the need for catheter placement and the accompanying risk of catheter-associated infections and other complications. A case study from Italy highlighted how these difficulties in management can be overcome with RZF. A patient with *C. glabrata* endocarditis with MIC elevated to fluconazole was a poor surgical candidate for valve replacement (20). Treatment was initiated with anidulafungin, and a venous catheter was placed, which subsequently became infected with *Enterococcus faecalis* requiring removal. RZF was administered through compassionate use approval for suppressive therapy. Valvular vegetations resolved on echocardiography and serial beta-D glucan levels down trended to negativity throughout the treatment course.

Additionally, caring for patients with candidemia is cost-intensive due to long hospital length of stays and intensive care unit (ICU) level care. RZF could potentially mitigate healthcare costs. A cost of care analysis in Germany examined the total cost related to candidemia cases. The study utilized the STRIVE finding of shorter ICU length of stay of 5 days to extrapolate potential savings of around €7100 per hospital case (21).

Emerging resistance has led to the investigation of the pharmacokinetics of echinocandins. Pharmacological strategies to prevent the development of resistance mechanisms and boost efficacy have been studied. Researchers have taken efforts to revisit the pharmacokinetics and pharmacodynamics of echinocandin dosing, as these evaluations were not commonplace during the original development of the daily dosed echinocandins (4). Anidulafungin, caspofungin, and micafungin all demonstrate concentration-dependent fungicidal activity *in vitro* (13, 22). Rezafungin demonstrated similar activity during *in vitro* analysis (23). A quantitative review showed fungicidal killing is correlated to AUC relative to MIC (3). Further efforts studied different dosing strategies to evaluate the effect of the shape of AUC:MIC ratio on efficacy in mouse models (23). Neutropenic mice were given disseminated candidiasis and treated with RZF either in a front-loaded dose or an equal dose divided twice weekly. Those given a front-loaded dose had greater efficacy in fungal killing than the divided dose arm of the study. These results suggest some barriers to efficacy could be surmounted by front loading. STRIVE and ReSTORE suggest a possible trend in a difference in rapid clearance and efficacy for RZF. Similarly, a high front-loading dose could also potentially lead to a higher barrier to resistance to RZF. Mutant prevention concentration (MPC) is the level a drug must attain to suppress subpopulations that persist beyond MIC levels. Finding the ideal MPC in fungal medications is a delicate balance between tolerability and efficacy. Comparison of the MPC values of micafungin and RZF against *Candida albicans* and *C. glabrata in vitro* found roughly equivalent MPC values (24). Later study showed that RZF’s unique pharmacokinetics demonstrated greater tissue penetration at levels greater than MPC compared to micafungin (25). Greater penetration led to greater degrees of reduction in the fungal burden of disease, further supporting the AUC:MIC ratio hypothesis. These findings in the context of RZF’s long half-life could confer a benefit in the prevention of the development of resistance patterns compared to other echinocandins (24). Both STRIVE and ReSTORE demonstrated a trend toward slightly faster time to negative blood cultures, which could serve to bolster this hypothesis.

As mentioned previously, FKS subunit mutations have been shown to cause echinocandins resistance in various *Candida* species, most notably *Candida glabrata* (26). FKS mutation along with increasing MIC values are associated with poor response to therapy and treatment failure (20). Murine models showed that RZF has equivalent or augmented efficacy at the reduction in fungal burden when compared to micafungin among caspofungin-resistant strains of *C. albicans* (24). Analysis of ReSTORE outcomes by *Candida* species showed *C. glabrata* isolates with FKS mutation demonstrated reduced *in vitro* susceptibility to RZF but maintained clinical success (18). These results serve to bolster the aforementioned pharmacokinetic benefits in overcoming resistance of RZF.

The increasing prevalence of *Candida auris* infections remains a public health concern (27). Over time, several geographically distinct clades have arisen, and cases have been documented on five continents. Mortality rates can often approach 60% (28). This species exhibits a high degree of resistance to azoles and also amphotericin B among selected strains. Micafungin demonstrated superior efficacy compared to azoles and amphotericin B *in vitro* (29). As a result, echinocandins are the standard empiric choice for *C. auris* infections. Despite this efficacy, multiple isolates have been shown to demonstrate echinocandin resistance (24, 29). *In vitro* data show that rezafungin has potent activity against a panel of variants of *Candida auris*, including those with azole and polyene resistance (30). Further work by the same research group demonstrated *in vivo* activity in mouse models. Neutropenic mice injected with *C. auris* treated with rezafungin had a superior reduction of colony counts in tissue compared to amphotericin B (28). RZF-treated mice also had a statistically significant reduction in fungal burden compared to micafungin in this study. The researchers speculate that RZF’s front-loaded dosing is the rationale behind this difference, hopefully providing a reliable solution to this emerging, resistant pathogen.

Another practical application could arise among patients with prolonged neutropenia and severe immunosuppression where RZF could potentially play a role in anti-fungal prophylaxis during periods of maximum vulnerability to invasive fungal infection. Patients with hematologic malignancy undergoing chemotherapy and/or hematopoietic stem cell transplantation have a high risk of fungal infections and high attributable mortality. Three of the most common fungal pathogens in these populations are *Candida* species, *Aspergillus* species, and *Pneumocystis* species (31). Standard prophylactic regimens include azoles and trimethoprim-sulfamethoxazole. While these agents are effective, they each have well-documented side effect burdens and drug-drug interactions. Echinocandins are generally not used as first-line prophylaxis given the need for daily dosing, need for long-term IV access, and their fungistatic activity against *Aspergillus* (25). *In vitro* studies show that RZF has activity against *Aspergillus flavus* and *A. fumigatus* similar to that of other echinocandins (32). Animal models showed good efficacy of RZF prevention against *Candida, Aspergillus*, and *Pneumocystis*. (25, 33) An immunocompromised mouse model demonstrated similar 6-week efficacy between standard care trimethoprim-sulfamethoxazole to RZF in the prevention of *Pneumocystis murina* infection (33). An ongoing phase III double-blind study (ReSPECT) will evaluate the efficacy toward the prevention of invasive fungal disease compared to standard azole and trimethoprim-sulfamethoxazole regimens in patients with allogeneic bone marrow transplantation. This study will evaluate a 13-week period of prophylaxis, assessing both non-inferiority and 90-day fungal-free survival (25). Utilizing this agent for prophylaxis could have a lower side effect burden and lessen medication interactions.

## CONCLUSION

The unique pharmacology of rezafungin yields enormous promise in the fight against growing anti-fungal resistance, as well as additional clinical possibilities extrapolated from clinical, *in vivo*, and *in vitro* data. Once-weekly dosing of rezafungin has been shown in randomized clinical trials to be as efficacious as standard therapy with daily dosing of conventional echinocandins. This agent boasts many of the beneficial properties of echinocandins, first and foremost being excellent tolerability and safety. RZF demonstrates a unique, extended half-life that offers numerous clinical possibilities to better utilize healthcare resources in treating azole-resistant fungal infections. The hypothesis that front-loading echinocandins could improve clearance of fungemia, therefore serving to better outcomes as well as preventing the development of resistance, is an exciting proposition supported by clinical and pre-clinical data. Further research is needed to examine this claim and better understand the mechanisms. The ongoing ReSPECT clinical trial could open an exciting new role for RZF in anti-fungal prophylaxis for patients with hematologic malignancy and other highly immunocompromised populations.
